# Efficacy and safety of taxanes combined with chemotherapy drugs in advanced triple negative breast cancer: A meta-analysis of 26 randomized controlled trials

**DOI:** 10.3389/fonc.2022.972767

**Published:** 2022-08-31

**Authors:** Qionglian Huang, Zubing Mei, Xianghui Han

**Affiliations:** ^1^ Institute of Chinese Traditional Surgery, Longhua Hospital Affiliated to Shanghai University of Traditional Chinese Medicine, Shanghai, China; ^2^ Department of Anorectal Surgery, Shuguang Hospital Affiliated to Shanghai University of Traditional Chinese Medicine, Shanghai, China

**Keywords:** triple negative breast cancer, taxane, combination therapy, efficacy, safety, meta-analysis

## Abstract

**Background:**

Researchers have demonstrated that the combined use of taxanes and chemotherapy drugs, especially paclitaxel-based treatment, appeared to clinically benefit on advanced triple negative breast cancer (TNBC). This meta-analysis aims to obtain the existent evidence on efficacy and safety for taxanes-based combination therapy to treat advanced TNBC.

**Methods:**

From 1991 to June 2022, seven databases (PubMed, Web of Science, Cochrane Library, Embase VIP, Wanfang, and CNKI databases) were comprehensively searched with no restricted language and region. The included randomized controlled trials (RCTs) compared taxanes-based combination therapy versus taxanes or other chemotherapy drugs. Statistical analysis was conducted using random-effect model, and the quality of RCTs was assessed using the tool of Cochrane Collaboration risk of bias.

**Results:**

Twenty-six RCTs with a total of 8,236 advanced TNBC patients were included. Compared with taxanes monotherapy, taxanes-based combination therapy significantly prolonged progression-free survival (HR=0.79, 95%CI=0.74–0.83, I^2^= 0.0%, *p*=0.000) and overall survival (HR=0.88, 95%CI=0.82–0.94, I^2^= 9.3%, *p*=0.000) and increased the risk of vomiting (RR=1.26, 95%CI=1.07–1.48) and diarrhea (RR=1.82, 95%CI=1.22–2.70, I^2^= 90.3%, *p*=0.003). No statistical differences were observed in complete response rate (CRR), objective response rate (ORR), disease control rate (DCR), and progressive disease (PD) indexes (CRR: RR=1.38, 95%CI=0.96–1.99; ORR: RR=1.20, 95%CI=0.73–1.98; DCR: RR=1.09, 95%CI=1.00–1.19; PD: RR=0.70, 95%CI=0.47–1.04). Compared with other chemotherapy drugs, taxanes *plus* other chemotherapy drugs significantly reduced the incidence of vomiting (RR=0.60, 95%CI=0.44–0.84, I^2^= 12.3%, *p*=0.002) and neutropenia (RR=0.58, 95%CI=0.35–0.96, I^2^= 73.0%, *p*=0.036) during the treatment period.

**Conclusions:**

Taxanes-based combination therapy is evidently effective and well-tolerated in advanced TNBC, indicating that it might be a recommended option for treating advanced TNBC patients to some extent.

**Systematic Review Registration:**

https://www.crd.york.ac.uk/PROSPERO/, identifier CRD42022337802.

## Introduction

Female breast cancer, with an assessed 2.3 million new cases and 0.68 million mortalities, has become the most common malignant tumor of global cancers in 2020 (1). Triple negative breast cancer (TNBC), regarded as a heterogeneous and aggressive breast cancer subtype and characterized by impaired expression of estrogen receptors, progesterone receptors, and human epidermal growth factor receptor 2, represents 10%–25% of breast cancers types and thus is strongly associated with poorer prognosis (2, 3). Furthermore, advanced TNBC usually leads to higher incidence of distant metastases such as bone, visceral, and central nervous system metastases within 5 years of diagnosis and causes high mortality afflicting on patients (4, 5). To date, there is no standard treatments for advanced TNBC, while chemotherapy was a recommended choice of treating TNBC (6–8).

Taxanes (i.e., nab-paclitaxel, paclitaxel, and docetaxel), are diterpenoid alkaloid compound with prominent antineoplastic activities. As the first-line chemotherapy drugs, taxanes were widely used in the treatment of advanced lung cancer, endometrial cancer, breast cancer, and ovarian cancer (9). US FDA approved taxanes for treating advanced or metastatic breast cancer in 2005 (10). Recently, the combination use of taxanes and other chemotherapy drugs, especially paclitaxel-based treatment, appears to be significantly beneficial on advanced TNBC patients (11, 12). A randomized clinical trial (RCT) reported that paclitaxel plus capivasertib therapy showed an improvement in progression-free survival and overall survival compared to paclitaxel monotherapy (13). Another RCT found that there were longer progression-free survival (PFS) and higher objective response rate (ORR) in advanced TNBC patients treated with nab–paclitaxel–carboplatin than gemcitabine–carboplatin (14). Paclitaxel combined with either bevacizumab or capecitabine also was set as therapy regimens for advanced TNBC, and the latter appeared to have better superiority in terms of progressive disease (15). Additionally, paclitaxel plus gemcitabine appeared less toxic than cisplatin plus gemcitabine totally when treating advanced TNBC (16). Thus, taxanes combined with other chemotherapy drugs were considered as the potential effective treatment choice based on the results of these studies.

However, to date, there is no clear evidence that taxanes plus other chemotherapy drugs benefits the advanced TNBC patients due to the limitation of small sample size included in these studies. Therefore, we summarize the date and relevant data for a comprehensive meta-analysis of all RCTs aiming to better elucidate the efficacy and safety of taxanes combined with chemotherapy drugs in advanced TNBC.

## Materials and methods

This meta-analysis was conducted in accordance with the Preferred Reporting Items for Systematic Reviews and Meta-analyses (17, 18) and registered at the International Prospective Register of Systematic Reviews (PROSPERO, CRD42022337802).

### Data sources and search strategy

Of no language or region restrictions, we searched PubMed, Cochrane Library, Web of Science, and Embase databases and three databases of China (CNKI, Wanfang, and VIP) from inception to February 20, 2022 systematically to recognize the full-text articles related to RCTs. We performed the following methodology to search the databases: using MeSH terms of “triple negative breast cancer” AND (“paclitaxel” OR “docetaxel”) AND (“metastasis” OR “advanced”) and free terms of them *plus* randomized controlled trials. A more detailed search strategy is available at **Supplementary Table S1**. Assessment to the eligible articles was performed by two reviewers (QH and XH) independently after reading the titles and abstracts of all articles.

### Inclusion and exclusion criteria

The identification for eligible literatures was carried out by using EndNote X9 software. Selection and assessment to the studies through different databases were conducted by two reviewers (QH and XH) independently according to PICOS criteria. The studies were included if they met the following criteria (18):

Participant: patients with age of more than 18 years and histologically confirmed advanced or metastatic TNBC (Eastern Cooperative Oncology Group performance status of 0–2).

Intervention: taxanes combined with other chemotherapy drugs (i.e., platinum, tabines, bevacizumab, and atezolizumab).

Comparator: taxanes or other chemotherapy drugs.

Outcomes: PFS, overall survival (OS), complete response rate (CRR), ORR, disease control rate (DCR), progressive disease (PD), and adverse events provided any analyzable data.

Study design: RCTs with either double-blind or multicenter design.

Exclusion criteria were the following: only abstract (19, 20), review (21), experimental research (22), case reports (23), non-RCTs (24), non-advanced TNBC (25), phase I trials (26), trials with improper control drugs (27), and no available data or duplicates (28, 29).

### Data extraction and quality assessment

Two reviewers (QH and XH) independently extracted data from eligible essays in terms of the following information according to the Cochrane Handbook guidelines: first author, study design, number of participants, median age, inclusion criteria, treatment duration, primary outcomes, and secondary outcomes. The participation of a third reviewer became a necessity when discrepancies occurred during the extraction of the data and information until consensus was realized.

The efficacy and safety of taxanes combination chemotherapy on advanced TNBC were appraised by PFS, OS, CRR, ORR, DCR, and PD, and the adverse events of taxanes combination chemotherapy were evaluated according to Response Evaluation Criteria in Solid Tumors (RECIST)1.1 and WHO grading criteria (30, 31). To fulfill credible conclusions for the reviewers, the Cochrane Collaboration risk of bias tool was used to analyze the data of random sequence generation, allocated concealment, detailed information of participants blinding, completion of outcome reporting, and selective publication for assessment to methodological quality of RCTs. Grading of Recommendations, Assessment, Development and Evaluations system (GRADE) was used to assess the quality of evidence for each outcomes (32).

### Definition of outcomes

PFS, OS, ORR, and total adverse events were selected as primary outcomes in this meta-analysis. Based on WHO general objective efficacy indicators of solid tumors or RECIST1.0 criteria (33), CRR, DCR, and PD were selected as secondary outcomes. PFS, the most common primary endpoint in cancer trials, is defined as the time from the date of initial treatment to the date of the first objective documentation of disease progression or the date of the last follow-up for patients who are still alive without disease progression or death without disease progression (34). OS, considered as the best therapeutic endpoint in tumor clinical trials, is interpreted as the time between randomization and death from any cause in a clinical trial (35). CRR is defined as the proportion of patients who achieved best overall response of confirmed complete responses (31). ORR is defined as the proportion of patients who achieved best overall response of confirmed complete responses and partial responses (31, 36). DCR, including cases of complete responses partial responses and stable disease, is the ratio of patients whose tumors shrink or remain stable for a certain period of time (31). PD means the sum diameter of lesions of patients increasing the sum of the largest diameter of lesions to at least 20% or greater or the emergence of a new lesion, which is often used to evaluate the aggravation of anti-tumor therapy in clinical trials (37). Adverse events from intervention and comparator drugs assessed in this article include total adverse events, anemia, vomiting, diarrhea, neutropenia, alopecia, and fatigue.

### Statistical synthesis and analysis

Statistical analysis was executed using Stata software (Version 12.0.) and random-effect model along with 95% confidence intervals (CIs) were used to analyze all quantitative data. For dichotomous variables, hazard ratio (HR) was used to appraise the indexes of PFS and OS. For effect variables, risk ratio (RR) was used to evaluate the indexes of ORR, DCR, PD, and adverse events. Data of each index were analyzed and presented by Forest plots; *p*<0.05 was considered as statistical significance. To explore potential resources, the clinical benefit indexes including PFS, OS, CRR, ORR, DCR, PD, and adverse events were highlighted by the conduction of subgroup analyses. Subgroup analysis was planned according to the types of control groups including other chemotherapy drugs or taxanes.

The between-study heterogeneity was assessed using Cochrane’s Q and I^2^ statistic as follows: 0%–40%, might not be important; 30%–60%, might represent moderate heterogeneity; 50%–90%, might represent substantial heterogeneity; and 75%–100%, considerable heterogeneity (38, 39). Publication bias was investigated visually according to the results of funnel plots and Egger’s test. When outcomes met more than 10 RCTs, the standard error of log (HR) and HR or log (RR) and RR were used to generate funnel plots. It is classified as publication bias if the results of Egger’s test are *p*< 0.05 and funnel plots are asymmetric.

## Results

### Search and selection of studies

**Figure 1:**
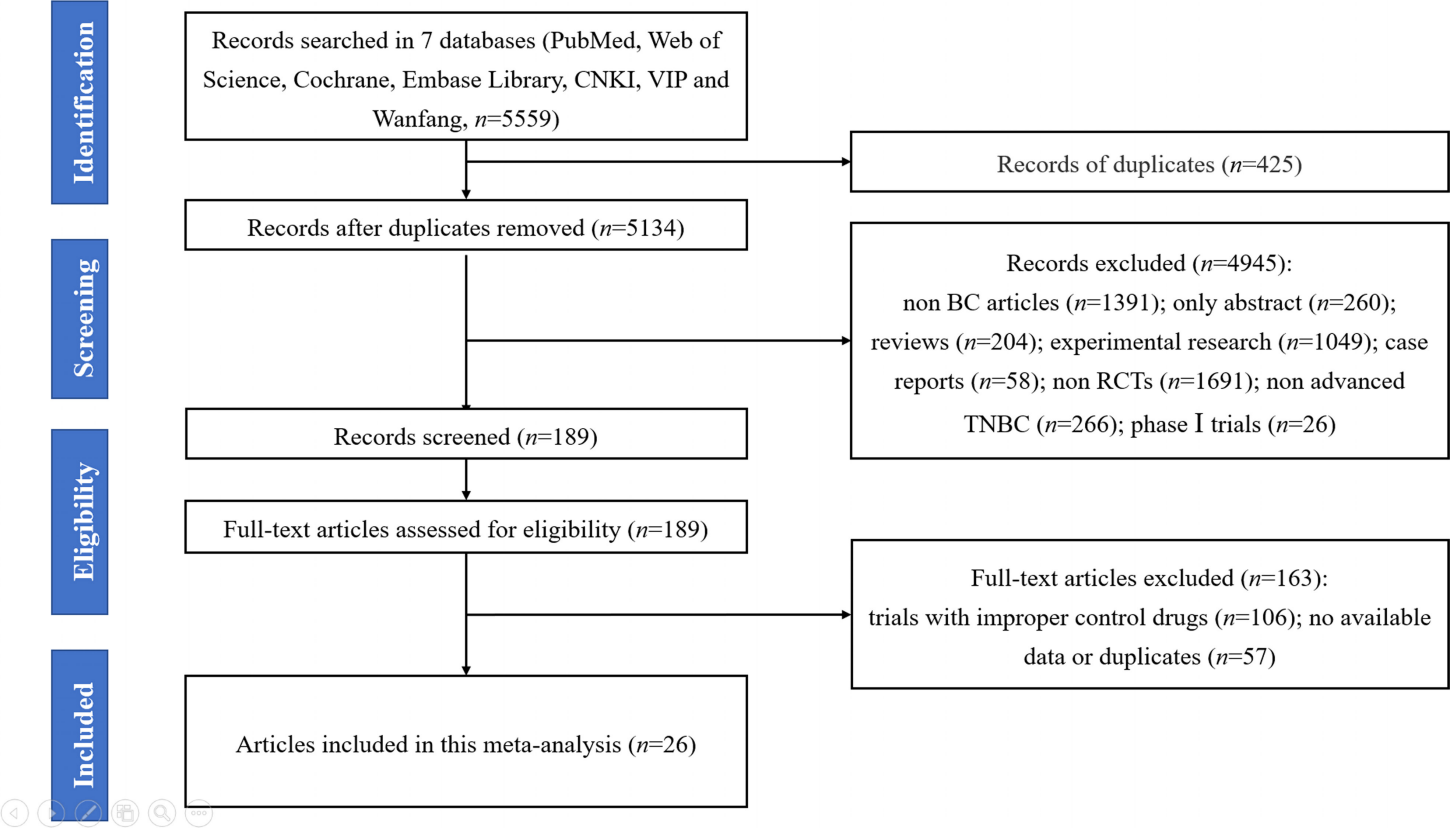
The PRISMA flowchart summarizing the process to identify randomized controlled trials for inclusion.

A flow diagram (**Figure 1**) presented the procedure on how we identified the articles in this meta-analysis. First, we collected 5,559 records by searching seven databases (PubMed, Web of Science, Cochrane Library, Embase VIP, Wanfang, and CNKI databases). Next, 5,370 records were excluded for some reasons (i.e., duplicates, non-breast cancer articles, only abstract, reviews, experimental research, case reports, non-RCTs, non-advanced TNBC, and phase I trials), and 189 full-text articles were considered as prospective eligibility. After further identification, 163 studies were excluded due to erroneous control agents and no available data or duplicates. Finally, 26 full-text RCTs were included in this meta-analysis (13–16, 40–61).

### Study characteristics

The fundamental characteristics of the 26 final included articles published in Chinese and English journals from 2011 to 2021 are summarized in **Supplementary Table S2**. These phase II or phase III trials involved 8,236 patients and were conducted in America, Europe, Asia, and Oceania. All patients histologically confirmed unresectable advanced or metastatic TNBC, ranging from 40.1 to 59.0 years of age.

Among these 26 RCTs, 12 were designed as double-blind or placebo-controlled (13, 42–44, 46, 50, 51, 53, 55–57, 60), 5 were designed as open-label or multicenter (14, 16, 40, 52, 61), and 9 were not described further in detail (15, 41, 45, 47–49, 54, 58, 59). The intervention arms in all trials were taxanes (nab-paclitaxel, 100 mg/m^2^; paclitaxel, 80–175 mg/m^2^; docetaxel, 75 mg/m^2^) plus other chemotherapy drugs (atezolizumab, 7 trials; carboplatin, 3 trials; bevacizumab, 3 trials; gemcitabine, 2 trials; cisplatinum, 2 trials; oxaliplatin, 2 trials; ipatasertib, 2 trials; capecitabine, 1 trial; tigatuzumab, 1 trial; capivasertib, 1 trial; reparixin, 1 trial; cobimetinib, 1 trial), while the control arms were taxanes (17 trials) or other chemotherapy drugs (9 trials). For primary outcomes, 19 trials assessed PFS and OS indexes of patients, 22 studies investigated ORR index of patients, and 12 trials observed the safety of drugs. For secondary outcomes, 6 trials reported CRR, 12 trials reported DC, and 14 trials reported PD were evaluated.

### Quality assessment and risk of bias

**Figure 2:**
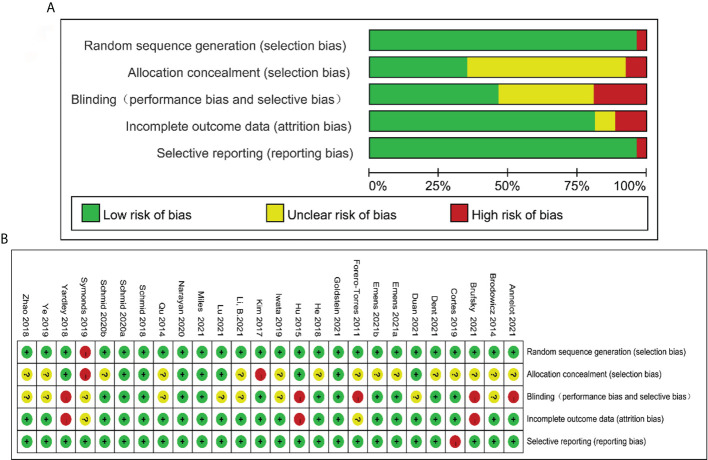
Risk of bias graph: reviewers’ judgments about each risk of bias item presented as percentages across all included studies **(A)**. Risk of bias summary: reviewers’ judgments about each risk of bias item for each included study according to the Cochrane Collaboration’s “Risk of Bias” tool, the green circle with “plus” sign low risk of bias information, the yellow circle with “question mark” sign representing unclear risk of bias information, and the red circle with “minus” sign representing high risk of bias information **(B)**.

The evaluation result of risk of bias is shown in **Figures 2A, B**. Of 26 eligible RCTs, 25 reported adequate random sequence generation, 9 covered allocation concealment, 12 performed double-blind way, 21 avoided incomplete outcome data, and 25 averted selective reporting bias.

The high-quality evidence with heterogeneity I^2^ was used as judgement of outcomes of clinical efficacy, including PFS, OS, and PD indexes, which ranged from 6.2% to 67.5%. CRR, ORR, and DCR indexes were judged as moderate-quality evidence with heterogeneity I^2^ ranging from 0% to 98.6%. Adverse effects such as diarrhea and alopecia were judged as high quality with heterogeneity I^2^ ranging from 6.2% to 87.8%, whereas total adverse events, vomiting, neutropenia, and fatigue were judged as moderate-quality evidence (heterogeneity I^2^= 14.0%–89.8%, **Supplementary Table S3**).

### Publication bias

**Figure 3:**
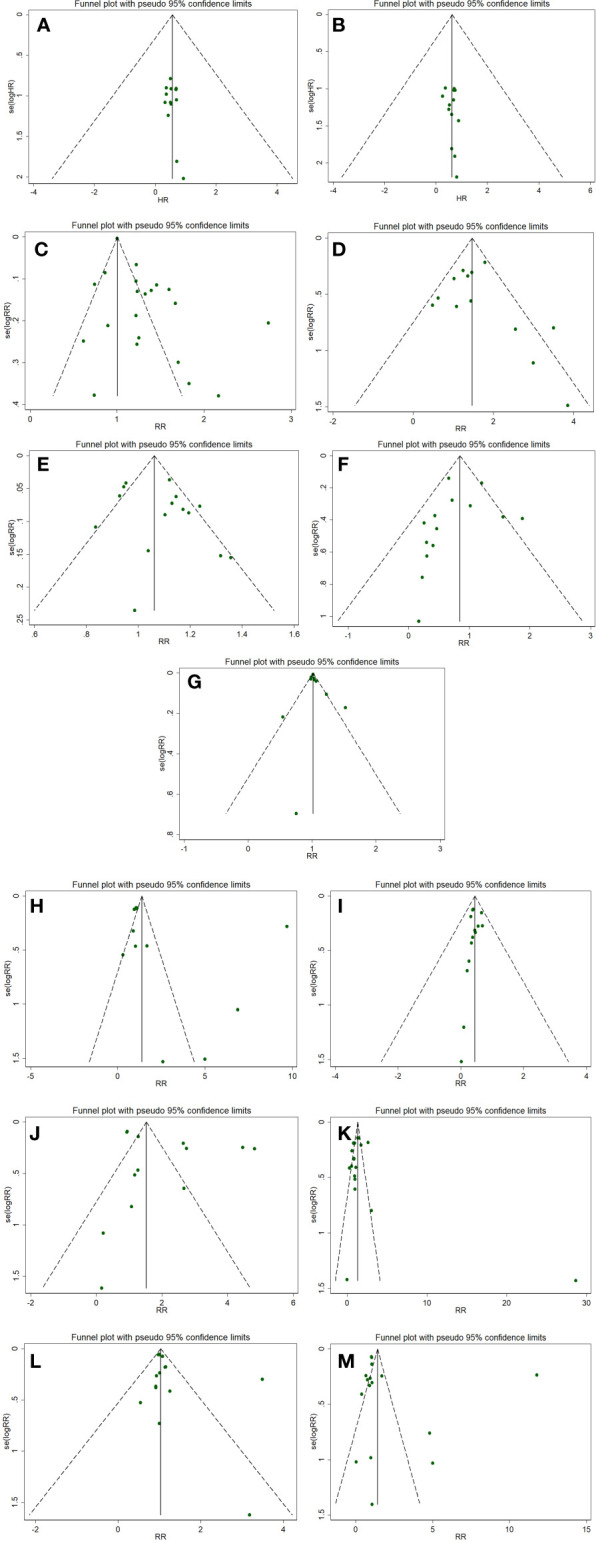
Funnel plots evaluating publication bias for following outcomes: PFS **(A)**, OS **(B)** OS, ORR **(C)**, CRR **(D)**, DCR **(E)**, PD **(F)**, total adverse events **(G)**, anemia **(H)**, vomiting **(I)**, diarrhea **(J)**, neutropenia **(K)**, alopecia **(L)**, and fatigue **(M)**.

The publication bias of the outcomes (≥10 RCTs) was evaluated by the performance of funnel plot and Egger’s test. The funnel plots revealed almost symmetric in 11 outcomes including PFS, OS, DCR, PD, total adverse events, anemia, vomiting, diarrhea, neutropenia, alopecia, and fatigue. The results of Egger’s test showed no statistical significance in the above indexes (PFS, *p*=0.492; OS, *p*=0.608; total adverse events, *p*=0.554; anemia, *p*=0.283; vomiting, *p*=0.629; diarrhea, *p*=0.174; neutropenia, *p*=0.315; alopecia, *p*=0.217; fatigue, *p*=0.435), which indicates no distinct publication bias in this meta-analysis. Notably, ORR and CRR indexes appeared potential publication bias (ORR, *p*=0.004; CRR, *p*=0.030, **Figures 3A–M**).

## Results of meta-analysis

### Primary outcomes

#### Progression-free survival

**Figure 4:**
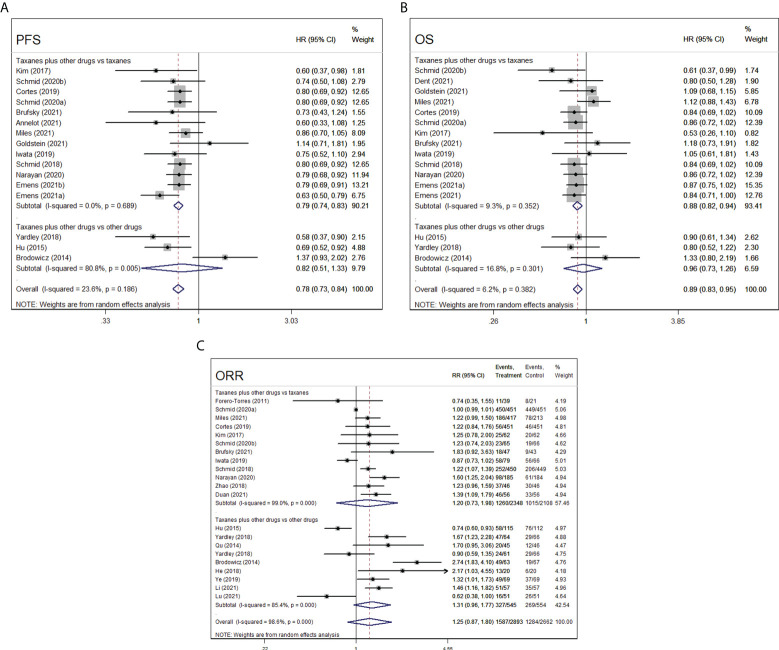
Forest plot of randomized controlled trials on taxanes combination therapy for primary outcomes: PFS **(A)**, OS **(B)**, and ORR **(C)**.

Sixteen RCTs reported about PFS index of taxanes plus other chemotherapy agents vs. taxanes or other chemotherapy agents (platinum, bevacizumab, and tabines). A total of 3,711 patients were included in taxanes combination groups and 3,494 patients in control groups. The overall results showed significant differences between the intervention groups and control groups (HR=0.78, 95%CI=0.73–0.84, I^2^= 23.6%, *p*=0.000). Subgroup analysis indicated that taxanes combination therapy was superior to taxanes monotherapy in terms of PFS (HR=0.79, 95%CI=0.74–0.83, I^2^= 0.0%, *p*=0.000), while no difference was observed between taxanes plus other chemotherapy agents and other chemotherapy agents (HR=0.82, 95%CI=0.51–1.33, I^2^= 80.0%, *p*=0.421, **Figure 4A**).

### Overall survival

With respect to OS, 16 RCTs included 3,758 patients who received taxanes plus other chemotherapy drugs and 3,541 patients who received taxanes or other chemotherapy drugs. The findings of the pooled data revealed that taxanes-combination therapy significantly prolonged OS of patients when comparing to taxanes monotherapy (HR=0.88, 95%CI=0.82–0.94, I^2^= 9.3%, *p*=0.000), whereas no significances were observed between taxanes combination groups and other agents combination groups (HR=0.96, 95%CI=0.73–1.26, I^2^= 16.8%, *p*=0.763, **Figure 4B**).

#### Objective response rate

In the 21 RCTs, 1,587 of 2,893 patients achieved an ORR in taxanes-based chemotherapy groups (intervention groups), and 1,284 of 2,662 patients achieved an ORR after the treatment of taxanes monotherapy or non-taxanes chemotherapy (control groups). The pooled 12 eligible studies reported taxanes alone, and the pooled nine eligible studies reported other chemotherapy drugs. Both pooled and subgroup analysis found that the intervention groups relatively have no distinct advantage to comparators (overall, RR=1.25, 95%CI=0.87–1.80, I^2^= 98.6%, *p*=0.227; taxanes plus other drugs vs. taxanes, RR=1.20, 95%CI=0.73–1.98, I^2^= 99.0%, *p*=0.474; taxanes plus other drugs vs. other drugs, RR=1.31, 95%CI=0.96–1.77, I^2^= 85.4%, *p*=0.084, **Figure 4C**)

#### Adverse events

All 26 RCTs reported the total adverse events and six mainly common adverse events (anemia, vomiting, diarrhea, neutropenia, alopecia, and fatigue) caused by different drugs, in which 1,566 of 1,673 patients in intervention groups and 1,489 of 1,620 patients in control groups suffered from adverse events.

**Figure 5:**
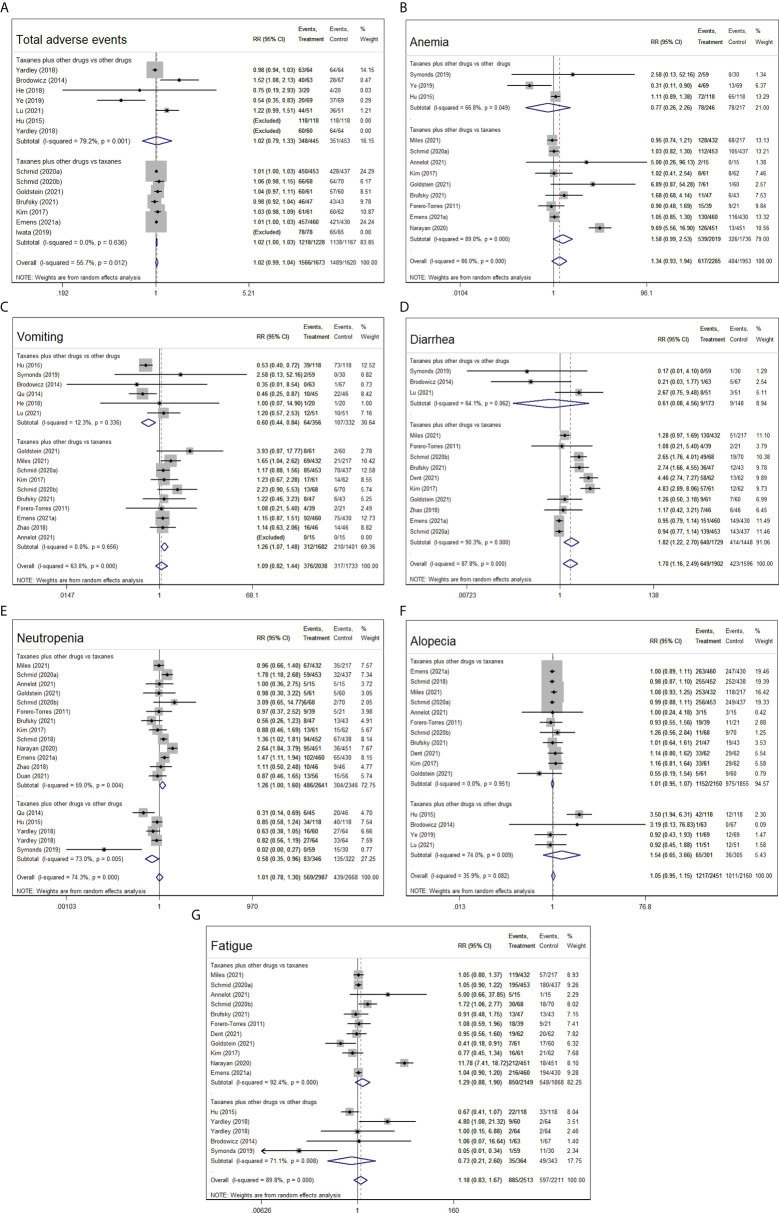
Forest plot of randomized controlled trials on taxanes combination therapy for adverse event: total adverse events **(A)**, anemia **(B)**, vomiting **(C)**, diarrhea **(D)**, neutropenia **(E)**, alopecia **(F)**, and fatigue **(G)**.

Overall and subgroup analyses of other chemotherapy drugs in control groups confirmed no statistical differences in the incidence risk of total adverse events between intervention groups (taxanes-based combination chemotherapy) and control groups (other chemotherapy drugs) (RR=1.02, 95%CI=0.99–1.04, I^2^= 55.7%, *p*=0.196; RR=1.02, 95%CI=0.79–1.33, I^2^= 79.2%, *p*=0.877). Nonetheless, the number of patients who accepted taxanes-based combination chemotherapy was obviously more than those who accepted taxanes monotherapy (RR=1.02, 95%CI=1–1.03, I^2^= 0.0%, *p*=0.004, **Figure 5A**).

Compared with taxanes monotherapy, taxanes-based combination therapy evidently increased the occurrence of vomiting (RR=1.26, 95%CI=1.07–1.48, I^2^= 0.0%, *p*=0.005) and diarrhea (RR=1.82, 95%CI=1.22–2.70, I^2^= 90.3%, *p*=0.003), whereas no differences were seen in the rest of the four adverse events. Compared with other chemotherapy drugs, taxanes plus other chemotherapy drugs obviously reduced the occurrence of vomiting (RR=0.60, 95%CI=0.44–0.84, I^2^= 12.3%, *p*=0.002) and neutropenia (RR=0.58, 95%CI=0.35–0.96, I^2^= 73.0%, *p*=0.036), whereas no significantly differences were observed in the other five adverse events (*p*>0.05, **Figures 5B–G**).

### Secondary outcomes

#### Complete response rate

**Figure 6:**
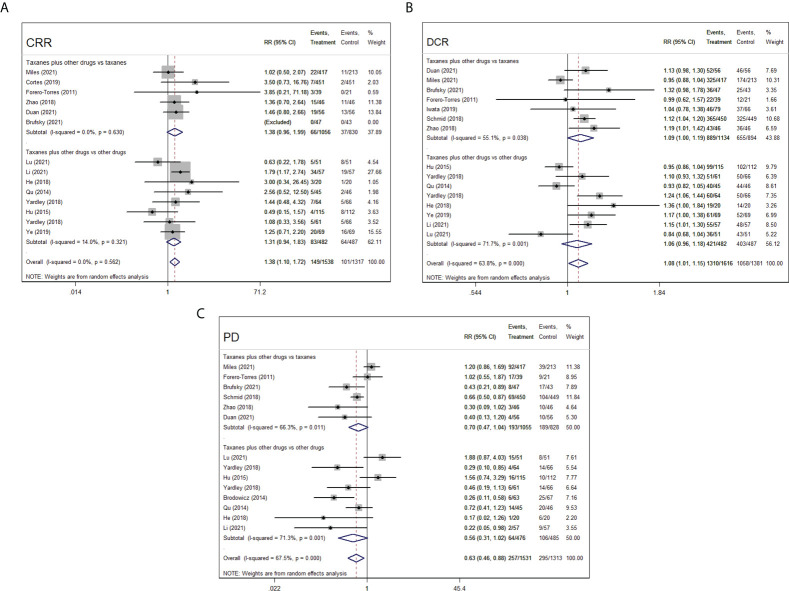
Forest plots of randomized controlled trials on taxanes combination therapy for secondary outcomes: CRR **(A)**, DCR **(B)**, and PD **(C)**.

Of 14 RCTs concerning CRR index, 6 RCTs (1,886 patients) with taxanes plus other chemotherapy drugs vs. taxanes only and 8 RCTs (969 patients) with taxanes plus other chemotherapy drugs vs. other chemotherapy drugs provided available data for CRR. In primary analysis, the taxanes-based combination treatment distinctly benefited the patients more in respect of CRR compared with the control arms (RR=1.38, 95%CI=1.10–1.72, I^2^= 0.0%, *p*=0.005). In secondary analysis, the number of patients in interventional arms who had complete response did not have any advantage over that in control arms (taxanes combination vs. taxanes, RR=1.38, 95%CI=0.96–1.99, I^2^= 0.0%, *p*=0.079; taxanes combination vs. non-taxanes drugs, RR=1.31, 95%CI=0.94–1.83, I^2^= 14.0%, *p*=0.110, **Figure 6A**).

#### Disease control rate

The included 15 RCTs in this meta-analysis covered DCR. The results from intervention groups showed superiority to stopping the deterioration of advanced TNBC in patients compared to control groups (RR=1.08, 95%CI=1.01–1.05, I^2^= 63.8%, *p*=0.027). The results of subgroup analysis suggested that there was insignificant superiority for taxanes combination therapy to increase the DCR in patients compared to taxanes monotherapy (RR=1.09, 95%CI=1.00-1.19, I^2^= 55.1%, *p*=0.053) or other chemotherapy drugs (RR=1.06 95%CI=0.96–1.18, I^2^= 63.8%, *p*=0.219, **Figure 6B**).

#### Progressive disease

In 14 RCTs, 257 of 1,531 (16.8%) patients accepting taxanes plus other chemotherapy drugs (intervention) and 295 of 1,313 (19.7%) patients accepting taxanes or other chemotherapy drugs (control) have undergone PD. The overall findings revealed that a lesser incidence of PD was seen in intervention groups than that in control groups (RR=0.63, 95%CI=0.46–0.88, I^2^= 67.5%, *p*=0.007). In further subgroup analysis, the results showed that there were insignificant differences in the number of patients in terms of PD between the medication regimens of taxanes plus other chemotherapy drugs and taxanes alone (RR=0.70, 95%CI=0.47–1.04, I^2^= 66.3%, *p*=0.075), and the same results were observed in the two groups of taxanes plus other chemotherapy drugs and other chemotherapy drugs (RR=0.56, 95%CI=0.31–1.02, I^2^= 71.3%, *p*=0.059, **Figure 6C**).

## Discussion

### Findings and interpretations

In this meta-analysis, we pooled the data of 26 RCTs, enrolling a total of 8,236 patients with advanced TNBC and compared taxanes-based combination therapies vs. taxanes or other chemotherapy drugs. Taken together, our results indicated that taxanes-based combination therapies had a significant beneficial effect on prolonging PFS and OS index of advanced TNBC patients compared with taxanes monotherapy. In clinics, taxanes plus other chemotherapy drugs (i.e., platinum, tabines, bevacizumab, and atezolizumab) was broadly applied in the treatment of advanced TNBC (15, 16, 61). Our results of meta-analysis found that taxanes plus bevacizumab could evidently improve ORR index and decrease PD index in patients compared to bevacizumab plus other drugs (*p*<0.05), but taxanes plus tabines or platinum revealed no statistical significance in therapeutic benefits compared to tabines or platinum plus other drugs (*p*>0.05). Additionally, taxanes *plus* other chemotherapy drugs showed more safe and well-tolerated by patients with advanced TNBC relative to other chemotherapy drugs. For example, taxanes plus other chemotherapy drugs and other chemotherapy drugs led to anemia in 31.7% and 35.9% of patients (16, 48, 49), vomiting in 18.0% and 32.2% of patients (15, 16, 41, 43, 49, 59), diarrhea in 5.2% and 6.1% of patients (15, 49, 59), neutropenia in 23.9% and 41.9% of patients (14–16, 41, 48), alopecia in 21.6% and 11.8% of patients (15, 16, 49, 59), and sfatigue in 35.2% and 27.0% of patients during the treatment period, respectively (14–16, 48). Simultaneously, indirectness of evidence, study design, publication bias, inconsistency in results, or data analysis objectively resulted in the potentially degradation of outcomes from included trials.

### Strengths and limitations

Strengths can be found in this meta-analysis as follows: first, to the best of our knowledge, this review is the first systematic investigation to explore the efficacy and safety of taxanes-based combination therapy for advanced or metastatic TNBC. Second, a large sample size including 8,236 patients in 26 RCTs published from 1991 to 2022 was assessed, with no restriction to language or region. Third, our meta-analysis summarized the existed recommendation to potential effect of taxanes on advanced TNBC, providing robustness for the results of studies. Fourth, subgroup analyses were made for the key outcomes basing on the combination of taxanes and other chemotherapy drugs versus taxanes alone or other chemotherapy drugs to minimize the possible selection bias and made the findings have great credibility. Fifth, our results became more reliable due to the execution of evaluating the quality of evidence for each individual outcome.

Nevertheless, several limitations in this meta-analysis should be taken into consideration. First, only 9 of 26 RCTs reported allocation concealment and 12 of 26 RCTs performed blinding to the measurement of the outcomes in our analysis, which might affect the accuracy of the results. Second, the choice to a random-effect model for all quantitative data in this meta-analysis might bring about more weight to smaller studies and wider confidence intervals, concealing potentially expanded effects of bias in these studies. Third, funnel plots and Egger’s test were not conducted to assess publication bias if the outcome was <10 RCTs included.

## Conclusion

In summary, the findings of this meta-analysis demonstrated that taxanes-based combination therapy is evidently effective to treat advance TNBC than taxanes monotherapy. Moreover, taxanes-based combination had a similar efficacy and fewer adverse reactions in comparison to other chemotherapy combination. Recent studies reported that the combination therapy of taxanes and new chemotherapy drugs (tigatuzumab, atezolizumab, and bevacizumab) was widely applied in clinical practice and presented more excellent therapeutical effects. Therefore, taxanes-based combination therapy, especially taxanes *plus* chemotherapy drugs, might become a recommended option to treat advance TNBC.

## Data availability statement

The raw data supporting the conclusions of this article will be made available by the authors, without undue reservation.

## Author contributions

XH and QH designed the study, collected and interpreted the data, and revised the article. QH performed the systematic literature search, analyzed the data, and wrote the original draft. XH, QH, and ZM contributed to the critical revision of the manuscript for important intellectual content. All authors contributed to the article and approved the submitted version.

## Acknowledgments

The authors thank all the authors of the original studies included in this meta-analysis. The research, authorship, and publication of this article were funded by the Natural Science Foundation of Shanghai, China (grant number: 20ZR1458600) and National Natural Science Foundation of China (grant number: 82174016).

## Conflict of interest

The authors declare that the research was conducted in the absence of any commercial or financial relationships that could be construed as a potential conflict of interest.

## Publisher’s note

All claims expressed in this article are solely those of the authors and do not necessarily represent those of their affiliated organizations, or those of the publisher, the editors and the reviewers. Any product that may be evaluated in this article, or claim that may be made by its manufacturer, is not guaranteed or endorsed by the publisher.

## Abbreviations

TNBC, triple negative breast cancer; PFS, progression-free survival; OS, overall survival, CRR: complete response rate; ORR, objective response rate; DCR, disease control rate; PD, progressive disease; HR, hazard ratio; RR, risk ratio; Cis, confidence intervals; RCTs, randomized controlled trials; RECIST, Response Evaluation Criteria in Solid Tumors.
