# Supplementary material for: Efficacy and safety of taxanes combined with chemotherapy drugs in advanced triple negative breast cancer: A meta-analysis of 26 randomized controlled trials
Source: Front Oncol. 2022 Aug 31;12:972767. doi: 10.3389/fonc.2022.972767 (PMC9471016; doi:10.3389/fonc.2022.972767)
Supplement: Supplementary file 1 [file Table_1.docx]

| **Library 1** | **PubMed** | |
| --- | --- | --- |
| **#** | **Search Details** | **Results** |
| #1 | "Triple Negative Breast Neoplasms"[MeSH Terms] | 7461 |
| #2 | (("ER-Negative PR-Negative HER2-Negative Breast Neoplasms OR Triple-Negative Breast Cancer OR Breast Cancer, Triple-Negative OR Breast Cancers, Triple-Negative OR Breast Cancer, Triple-Negative OR Triple-Negative Breast Cancers OR Triple-Negative Breast Neoplasm OR Breast Neoplasm, Triple-Negative OR Breast Neoplasms, Triple-Negative OR Triple Negative Breast Neoplasm OR Triple-Negative Breast Neoplasms OR ER-Negative PR-Negative HER2-Negative Breast Cancer OR ER Negative PR Negative HER2 Negative Breast Cancer OR Triple Negative Breast Cancer OR Breast Neoplasm")[All Fields]) | 382,501 |
| #3 | #1 OR #2 | 382,501 |
| #4 | "Docetaxel"[MeSH Terms] | 11553 |
| #5 | "Paclitaxel"[MeSH Terms] | 29,074 |
| #6 | (("Taxane OR Taxol OR PTX OR Docetaxel OR Anzatax OR NSC 125973 OR NSC125973 OR Taxol A OR Bris Taxol OR Taxol, Bris OR Paclitaxel, 4 alpha Isomer OR Paxene OR Praxel OR 7 epi Taxol or Onxol") [All Fields]) | 63,239 |
| #7 | #4 OR #5 OR #6 | 63,239 |
| #8 | (("advanced Triple Negative Breast Cancer or cancer cell metastasis or cancer metastasis or carcinoma metastasis or metastases or metastatic type or metastasis formation or metastatic cancer or metastatic cancers or metastatic carcinoma or metastatic carcinomas or metastatic disease or metastatic tumor or metastatic tumors or metastatic tumour or metastatic tumours or neoplasm metastasis or sarcoma metastasis or secondary cancer or secondary carcinoma or tumor metastasis or tumor migration or tumour metastasis or tumour migration or hematogenous metastasis or Invasion and metastasis)") [All Fields]) | 827,969 |
| #9 | (("randomized controlled trial OR controlled clinical trial’ OR placebo OR randomized OR trial OR randomly OR group OR Double blind OR Single blind OR random OR controlled") [All Fields] | 6,837,950 |
| #10 | #3 AND #7 AND #8 AND #9 | 2757 |

| **Library 2** | **Embase** | |
| --- | --- | --- |
| **#** | **Search Details** | **Results** |
| #1 | triple negative breast cancer'/exp | 27892 |
| #2 | (‘ER-Negative PR-Negative HER2-Negative Breast Neoplasms’:ab,ti OR ‘Triple-Negative Breast Cancer’:ab,ti OR ‘Breast Cancer, Triple-Negative’:ab,ti OR ‘Breast Cancers, Triple-Negative’:ab,ti OR ‘Breast Cancer, (Triple-Negative’:ab,ti OR ‘Triple-Negative Breast Cancers’:ab,ti OR ‘Triple-Negative Breast Neoplasm’:ab,ti OR ‘Breast Neoplasm, Triple-Negative’:ab,ti OR ‘Breast Neoplasms, Triple-Negative’:ab,ti OR ‘Triple Negative Breast Neoplasm’:ab,ti OR ‘Triple-Negative Breast Neoplasms’:ab,ti OR ‘ER-Negative PR-Negative HER2-Negative Breast Cancer’:ab,ti OR ‘ER Negative PR Negative HER2 Negative Breast Cancer’:ab,ti OR ‘Triple Negative Breast Cancer’:ab,ti OR ‘Breast Neoplasm’) AND [humans]/lim | 18953 |
| #3 | #1 OR #2 | 30150 |
| #4 | docetaxel'/exp | 65401 |
| #5 | paclitaxel'/exp | 118940 |
| #6 | ('taxol':ab,ti OR 'ptx':ab,ti OR 'docetaxel':ab,ti OR 'anzatax':ab,ti OR 'nsc 125973':ab,ti OR 'nsc125973':ab,ti OR 'taxol a':ab,ti OR 'bris taxol':ab,ti OR 'taxol, bris':ab,ti OR 'paclitaxel, 4 alpha isomer':ab,ti OR 'paxene':ab,ti OR 'praxel':ab,ti OR '7 epi taxol':ab,ti OR 'onxol':ab,ti) AND [humans]/lim | 38976 |
| #7 | #3 OR #4 OR #5 | 165432 |
| #8 | 'metastasis'/exp | 730233 |
| #9 | ('advanced triple negative breast cancer':ab,ti OR'cancer cell metastasis':ab,ti OR 'cancer metastasis':ab,ti OR 'carcinoma metastasis':ab,ti OR 'metastases':ab,ti OR 'metastasic type':ab,ti OR 'metastasis formation':ab,ti OR 'metastatic cancer':ab,ti OR 'metastatic cancers':ab,ti OR 'metastatic carcinoma':ab,ti OR 'metastatic carcinomas':ab,ti OR 'metastatic disease':ab,ti OR 'metastatic tumor':ab,ti OR 'metastatic tumors':ab,ti OR 'metastatic tumour':ab,ti OR 'metastatic tumours':ab,ti OR 'neoplasm metastasis':ab,ti OR 'sarcoma metastasis':ab,ti OR 'secondary cancer':ab,ti OR 'secondary carcinoma':ab,ti OR 'tumor metastasis':ab,ti OR 'tumor migration':ab,ti OR 'tumour metastasis':ab,ti OR 'tumour migration':ab,ti OR 'hematogenous metastasis':ab,ti OR 'invasion and metastasis':ab,ti) AND [humans]/lim | 325878 |
| #10 | #8 OR #9 | 800176 |
| #11 | 'randomized controlled trial':ab,ti OR 'controlled clinical trial':ab,ti OR 'placebo':ab,ti OR 'randomized':ab,ti OR 'trial':ab,ti OR 'randomly':ab,ti OR 'group':ab,ti OR 'double blind':ab,ti OR 'single blind':ab,ti OR 'random':ab,ti OR 'controlled':ab,ti | 4464726 |
| #12 | #3 AND #7 AND #10 AND #11 | 471 |

| **Library 3** | **Cochrane** | |
| --- | --- | --- |
| **#** | **Search Details** | **Results** |
| #1 | MeSH descriptor: [triple negative Breast neoplasms] explode all trees | 333 |
| #2 | (ER-Negative PR-Negative HER2-Negative Breast Neoplasms OR Triple-Negative Breast Cancer OR Breast Cancer, Triple-Negative OR Breast Cancers, Triple-Negative OR Breast Cancer, Triple-Negative OR Triple-Negative Breast Cancers OR Triple-Negative Breast Neoplasm OR Breast Neoplasm, Triple-Negative OR Breast Neoplasms, Triple-Negative OR Triple Negative Breast Neoplasm OR Triple-Negative Breast Neoplasms OR ER-Negative PR-Negative HER2-Negative Breast Cancer OR ER Negative PR Negative HER2 Negative Breast Cancer OR Triple Negative Breast Cancer OR Breast Neoplasm):ti,ab,kw | 19109 |
| #3 | #1 OR #2 | 19113 |
| #4 | MeSH descriptor: [Docetaxel] explode all trees | 2247 |
| #5 | MeSH descriptor: [Paclitaxel] explode all trees | 3738 |
| #6 | (Taxol OR PTX OR Docetaxel OR Anzatax OR NSC 125973 OR NSC125973 OR Taxol A OR Bris Taxol OR Taxol, Bris OR Paclitaxel, 4 alpha Isomer OR Paxene OR Praxel OR 7 epi Taxol or Onxol):ti,ab,kw | 8918 |
| #7 | #4 OR #5 OR #6 | 12095 |
| #8 | MeSH descriptor: [Neoplasm Metastasis] explode all trees | 5413 |
| #9 | (advanced triple negative breast cancer or cancer cell metastasis or cancer metastasis or carcinoma metastasis or metastases or metastatic type or metastasis formation or metastatic cancer or metastatic cancers or metastatic carcinoma or metastatic carcinomas or metastatic disease or metastatic tumor or metastatic tumors or metastatic tumour or metastatic tumours or neoplasm metastasis or sarcoma metastasis or secondary cancer or secondary carcinoma or tumor metastasis or tumor migration or tumour metastasis or tumour migration or hematogenous metastasis or Invasion and metastasis):ti,ab,kw | 75819 |
| #10 | #8 OR #9 | 75941 |
| #11 | (randomized controlled trial OR controlled clinical trial OR placebo OR randomized OR trial OR randomly OR group OR Double blind OR Single blind OR random OR controlled):ti,ab,kw | 1522860 |
| #12 | #3 AND #7 AND #10 AND #11 | 1087 |

| **Library 4** | **Web of science** | |
| --- | --- | --- |
| **#** | **Search Details** | **Results** |
| #1 | ((TI=(ER-Negative PR-Negative HER2-Negative Breast Neoplasms OR Triple-Negative Breast Cancer OR Breast Cancer, Triple-Negative OR Breast Cancers, Triple-Negative OR Breast Cancer, Triple-Negative OR Triple-Negative Breast Cancers OR Triple-Negative Breast Neoplasm OR Breast Neoplasm, Triple-Negative OR Breast Neoplasms, Triple-Negative OR Triple Negative Breast Neoplasm OR Triple-Negative Breast Neoplasms OR ER-Negative PR-Negative HER2-Negative Breast Cancer OR ER Negative PR Negative HER2 Negative Breast Cancer OR Triple Negative Breast Cancer OR Breast Neoplasm)) OR AB=(ER-Negative PR-Negative HER2-Negative Breast Neoplasms OR Triple-Negative Breast Cancer OR Breast Cancer, Triple-Negative OR Breast Cancers, Triple-Negative OR Breast Cancer, Triple-Negative OR Triple-Negative Breast Cancers OR Triple-Negative Breast Neoplasm OR Breast Neoplasm, Triple-Negative OR Breast Neoplasms, Triple-Negative OR Triple Negative Breast Neoplasm OR Triple-Negative Breast Neoplasms OR ER-Negative PR-Negative HER2-Negative Breast Cancer OR ER Negative PR Negative HER2 Negative Breast Cancer OR Triple Negative Breast Cancer OR Breast Neoplasm)) OR TS=(Triple Negative Breast Cancer) | 28347 |
| #2 | (((TI= (Taxol OR PTX OR Docetaxel OR Anzatax OR NSC 125973 OR NSC125973 OR Taxol A OR Bris Taxol OR Taxol, Bris OR Paclitaxel, 4 alpha Isomer OR Paxene OR Praxel OR 7 epi Taxol or Onxol)) OR AB= (Taxol OR PTX OR Docetaxel OR Anzatax OR NSC 125973 OR NSC125973 OR Taxol A OR Bris Taxol OR Taxol, Bris OR Paclitaxel, 4 alpha Isomer OR Paxene OR Praxel OR 7 epi Taxol or Onxol)) OR TS=(Docetaxel)) OR TS=(Paclitaxel) | 92,366 |
| #3 | ((TI=(‘advanced Triple Negative Breast Cancer’ or ‘cancer cell metastasis’ or ‘cancer metastasis’ or ‘carcinoma metastasis’ or ‘metastases’ or ‘metastatic type’ or ‘metastasis formation’ or ‘metastatic cancer’ or ‘metastatic cancers’ or ‘metastatic carcinoma’ or ‘metastatic carcinomas’ or ‘metastatic disease’ or ‘metastatic tumor’ or ‘metastatic tumors’ or ‘metastatic tumour’ or ‘metastatic tumours’ or ‘neoplasm metastasis’ or ‘sarcoma metastasis’ or ‘secondary cancer’ or ‘secondary carcinoma’ or ‘tumor metastasis’ or ‘tumor migration’ or ‘tumour metastasis’ or ‘tumour migration’ or ‘hematogenous metastasis’ or ‘Invasion and metastasis’)) OR AB=(‘advanced Triple Negative Breast Cancer’ or’ cancer cell metastasis’ or ‘cancer metastasis’ or ‘carcinoma metastasis’ or ‘metastases’ or ‘metastatic type’ or ‘metastasis formation’ or ‘metastatic cancer’ or ‘metastatic cancers’ or ‘metastatic carcinoma’ or ‘metastatic carcinomas’ or ‘metastatic disease’ or ‘metastatic tumor’ or ‘metastatic tumors’ or ‘metastatic tumour’ or ‘metastatic tumours’ or ‘neoplasm metastasis’ or ‘sarcoma metastasis’ or ‘secondary cancer’ or ‘secondary carcinoma’ or ‘tumor metastasis’ or ‘tumor migration’ or ‘tumour metastasis’ or ‘tumour migration’ or ‘hematogenous metastasis’ or ‘Invasion and metastasis’)) OR TS=(metastasis) | 650,008 |
| #4 | (TI= (randomized controlled trial OR controlled clinical trial OR placebo OR randomized OR trial OR randomly OR group OR Double blind OR Single blind OR random OR controlled)) OR AB= (randomized controlled trial OR controlled clinical trial OR placebo OR randomized OR trial OR randomly OR group OR Double blind OR Single blind OR random OR controlled) | 12,043,467 |
| #5 | #1 AND #2 AND #3 AND #4 | 533 |

| **Library 5** | **CNKI** | |
| --- | --- | --- |
| **#** | **Search Details** | **Results** |
| #1 | MeSH= ‘Triple Negative Breast Cancer’ or TI= ‘Triple Negative Breast Cancer’ or AB= ‘Triple Negative Breast Cancer’ |  |
| #2 | MeSH= ‘Paclitaxel’ or TI= ‘Paclitaxel’ or AB= ‘Paclitaxel’ or SU%= ‘Docetaxel’ or TI= ‘Docetaxel’ or AB= ‘Docetaxel’ |  |
| #3 | MeSH= ‘Metastasis’ or TI= ‘Metastasis’ or AB= ‘Metastasis’ or SU%= ‘Advanced’ or TI= ‘Advanced’ or AB= ‘Advanced’ |  |
| #4 | MeSH= ‘Controlled’ or TI= ‘Controlled’ or AB= ‘Controlled' |  |
| #5 | #1 AND #2 AND #3 AND #4 | 22 |

| **Library 6** | **VIP** | |
| --- | --- | --- |
| **#** | **Search Details** | **Results** |
| #1 | TI= Triple Negative Breast Cancer or AB= Triple Negative Breast Cancer |  |
| #2 | TI= (Paclitaxel or Docetaxel) or AB= (Paclitaxel or Docetaxel) |  |
| #3 | TI= (Metastasis or Advanced’) or AB= (Metastasis or Advanced’) |  |
| #4 | TI= (Metastasis or Advanced) or AB= (Metastasis or Advanced’) |  |
| #5 | #1 AND #2 AND #3 AND #4 | 318 |

| **Library 7** | **Wanfang** | |
| --- | --- | --- |
| **#** | **Search Details** | **Results** |
| #1 | Title: "Triple Negative Breast Cancer" or Abstract: "Triple Negative Breast Cancer" |  |
| #2 | （Title: "Paclitaxel" or Abstract: "Paclitaxel" or Title: "Docetaxel" or Abstract: "Docetaxel"） |  |
| #3 | （Title: "Metastasis" or Abstract: "Metastasis" or Title: "Advanced" or Abstract: "Advanced"） |  |
| #4 | （Title: "controlled" or Abstract: "controlled"） |  |
| #5 | Secondary research: Triple Negative Breast Cancer, then searched ‘Metastasis or Advanced’ in results |  |
| #6 | #1 AND #2 AND #3 AND #4 AND #5 | 371 |
