# Supplementary material for: Efficacy and safety of taxanes combined with chemotherapy drugs in advanced triple negative breast cancer: A meta-analysis of 26 randomized controlled trials
Source: Front Oncol. 2022 Aug 31;12:972767. doi: 10.3389/fonc.2022.972767 (PMC9471016; doi:10.3389/fonc.2022.972767)
Supplement: Supplementary file 2 [file Table_2.docx]

**Supplementary Table 2. Characteristics of 26 included randomized controlled trials.**

| **First author**  **year** | | | **Study design** | **Number of participants** | **Median age (year)** | **Inclusion criteria** | **Treatment duration** | | **Study groups** | | **Primary outcomes** | | **Secondary outcomes** |
| --- | --- | --- | --- | --- | --- | --- | --- | --- | --- | --- | --- | --- | --- |
| Forero-Torres  2011 | An open label, randomized, phase II trial | | | Intervention: 42  Control:  22 | Intervention:  51 (32-72)  Control:  50.5 (34-75) | Women ≥ 18 years old histologically confirmed measurable mTNBC, with ECOG PS 0-2, adequate organ and bone marrow function, have no prior chemotherapy for metastatic disease and prior therapy with taxanes for metastatic disease were permitted | Treatment continued until disease progression or unacceptable toxicity | | Nab-paclitaxel (100 mg/m^2^,) + tigatuzumab (10 mg/kg loading dose followed by 5 mg/kg every other week) *vs* nab-paclitaxel (100 mg/m^2^) + placebo | | ORR | | PFS, DOR, CBR and safety |
| Qu  2014 | A randomized trial | | | Intervention: 45  Control:  46 | Intervention: 40.20±2.31  Control:  40.10±2.42 | Patients > 18 years old histologically confirmed mTNBC, with measurable specimen, life expectancy> 6 months, have prior (neo) adjuvant chemotherapy was permitted if completed> 3 months before the first dose | Treatment continued until disease progression | | Nab-paclitaxel (135 mg/m^2^ IV~~)~~ + carboplatin (5 mg/(mL**·**min) *vs* vinorelbine (25 mg/m^2^) + cisplatinum (25 mg/m^2^) | | PFS, OS | | ORR, DCR, quality of life |
| Brodowicz  2014 | A randomized  phase III trial | | | Intervention: 63  Control:  67 | Intervention: 54 (29-84)  Control:  56 (28-77) | Patients > 18 years old histologically confirmed mTNBC, with ECOG PS 0-2, have no prior chemotherapy for metastatic disease | NR | | Paclitaxel (90 mg/m^2^) + bevacizumab (10 mg/kg) *vs* capecitabine (1000 mg/m^2^, b.i.d.) + bevacizumab (15 mg/kg) | | PFS, OS | | ORR |
| Hu  2015 | A prospective, open-label, multicenter, randomized, phase Ⅲ trial | | | Intervention: 118  Control:  118 | Intervention: 48 (43-55)  Control:  47 (42-57) | Women histologically confirmed measurable mTNBC, with ECOG PS 0-1, have no prior chemotherapy for metastatic disease and prior therapy with taxanes for metastatic disease was permitted | Treatment continued until disease progression or death | | Paclitaxel (175 mg/m²) + gemcitabine (1250 mg/m²) *vs* cisplatin (75 mg/m²) + gemcitabine (1250 mg/m²) | | PFS | | OS, ORR and safety |
| Kim  2017 | A randomized, placebo-controlled, double-blind, phase II trial | | | Intervention: 62  Control:  62 | Intervention: 54 (44-63) Control:  53 (45-63) | Women ≥ 18 years old histologically confirmed measurable, locally advanced or mTNBC, with ECOG PS 0/1, adequate organ, have no prior chemotherapy for metastatic disease and prior therapy with taxanes for metastatic disease were permitted | Treatment continued until disease progression, intolerable toxicity, or withdrawal of consent | | Paclitaxel (80 mg/m²) + ipatasertib (400 mg/d) *vs* paclitaxel (80 mg/m²) + placebo | | PFS | | ORR, DOR, OS, efficacy, safety, PROs, quality of life |
| Schmid  2018 | A randomized, international, placebo-controlled double-blind, phase III trial | | | Intervention: 451  Control:  451 | Intervention: 50 (20-82) Control:  56 (26-86) | Patients > 18 years old histologically confirmed unresectable locally advanced or mTNBC, with measurable specimen, ECOG PS 0/1, life expectancy > 3 months, qualified inspection indicators, have no contraindications for taxanes monotherapy and prior (neo) adjuvant chemotherapy was not permitted | Treatment continued until disease progression or an unacceptable level of toxic effects occurred | | Nab-paclitaxel (100 mg/m^2^) + atezolizumab (840 mg) *vs* nab-paclitaxel (100 mg/m^2^) + placebo (840 mg) | | PFS, OS | | ORR, DOR, safety |
| Zhao  2018 | A randomized trial | | | Intervention: 46  Control:  46 | Intervention: 47.63±2.47  Control: 46.25±1.88 | Women ≥ 18 years old histologically confirmed advanced TNBC, with life expectancy> 6 months, ≥1 measurable lesions, had no contraindications to chemotherapy | NR | | Paclitaxel (135-175 mg/m^2^) + carboplatin (5 mg/(mL·min) *vs* paclitaxel (135-175 mg/m^2^) | | PFS, OS | | CRR, ORR, DCR |
| He  2018 | A double-blinded randomized trial | | | Intervention: 20  Control:  20 | Intervention: 43.6±2.5 Control: 43.1±2.6 | Patients ≥ 18 years old histologically confirmed advanced TNBC, with life expectancy> 3 months, have Karnofsky grade＞80 | Treatment continued until disease progression | | Docetaxel (75 mg/m^2^) + cisplatinum (25 mg/m^2^) *vs* vinorelbine (25 mg/m^2^) + cisplatinum (25 mg/m^2^) | | PFS, OS | | CRR, ORR, DCR |
| Yardley  2018 | An open-label, multicenter, randomized, phase II trial | | | G/C: 66  nab-P/G: 61  nab-P/C: 64 | P/C: 55  P/G:53  G/C: 59 | Women ≥ 18 years histologically confirmed measurable mTNBC, with measurable disease, ECOG PS 0-1, prior adjuvant or neoadjuvant anthracycline therapy (unless not indicated by physician), have received no prior cytotoxic chemotherapy | NR | | Nab-paclitaxel (125 mg/m^2^) + gemcitabine (1000 mg/m^2^);  nab-paclitaxel (125 mg/m^2^) + carboplatin (area under the curve 2); gemcitabine (1000 mg/m^2^) + carboplatin (area under the curve 2) | | PFS | | OS and safety |
| Ye  2019 | A randomized trial | | | Intervention: 69  Control:  69 | Intervention:  49.05±5.06  Control:  47.02±15.05 | Patients with histologically confirmed advanced TNBC, have no contraindications for chemotherapy | NR | | Paclitaxel (70 mg/m^2^) + cisplatinum (70 mg/m^2^) *vs* gemcitabine (1000 mg/m^2^) + cisplatinum (70 mg/m^2^) | | ORR | | CRR, DCR |
| Iwata  2019 | | A global phase Ⅲ trial | | Intervention: 79  Control:  66 | NR | Patients, with ECOG PS 0-1 and tumor tissue for PD-L1, have histologically documented advanced TNBC | | Treatment continued until disease progression | | Nab-paclitaxel (100 mg/m^2^) + atezolizumab (840 mg) *vs* nab-paclitaxel (100 mg/m^2^) + placebo (840 mg) | | PFS, OS | ORR, DOR |
| Cortes  2019 | | A randomized, placebo-controlled,  double-blind, phase Ⅲ trial | | Intervention: 451  Control:  451 | NR | Patients histologically confirmed locally recurrent, inoperable or mTNBC, with ECOG PS 0/1, life expectancy≥ 12 weeks, adequate hematologic and organ function, have received prior anthracycline and taxane therapy in the (neo) adjuvant setting and had completed any prior (neo) adjuvant chemotherapy for early breast cancer> 12 months starting the trial | | Treatment continued until disease progression, unacceptable toxicity or patient or physician decision | | Nab-paclitaxel + atezolizumab *vs* nab-paclitaxel + placebo | | OS | PFS, OS, ORR, DOR, CBR, patient-reported outcomes (PROs), safety and pharmacokinetics |
| Symonds  2019 | A phase II trial | | | Intervention: 59  Control:  30 | Intervention and Control: 54.9 (33-83) | Patients with mTNBC, had either measurable disease by RECIST criteria or non-measurable disease with rising serum CA15-3 or CA27.29 or CEA documented by two consecutive measurements taken at least 14 days apart, adequate organ function, were not pregnant and had no contraindications to bevacizumab | Treatment continued until disease progression | | Paclitaxel (100 mg/m^2^) + bevacizumab (10 mg/kg) *vs* bevacizumab (10 mg/kg) + erlotinib (150 mg PO daily) | | PFS | | ORR, OS and adverse events |
| Schmid  2020a | A randomized, placebo-controlled, double-blind, phase Ⅲ trial | | | Intervention: 451  Control:  451 | Intervention: 55 (46–64) Control:  56(47–65) | Patients histologically confirmed, unresectable, locally advanced or mTNBC, with ECOG PS 0-1 and life expectancy> 12 weeks, measurable specimen for PD-L, have no prior systemic therapy for advanced TNBC | Treatment continued until disease progression or unacceptable toxicity | | Nab-paclitaxel (100 mg/m^2^) + atezolizumab (840 mg) *vs* nab-paclitaxel (100 mg/m^2^) + placebo (840 mg) | | PFS, OS | | ORR, CBR, DOR, safety, quality of life |
| Schmid  2020b | A randomized, placebo-controlled, double-blind, phase II trial | | | Intervention: 68  Control:  70 | Intervention: 55.2 (48.2-61.4)  Control:  51.9 (40.8-60.7) | Patients histologically confirmed mTNBC, with evaluable or measurable disease, have no prior (neo)adjuvant chemotherapy and have completed taxanes treatment <12 months | Treatment continued until disease progression | | Paclitaxel (90 mg/m^2^) + capivasertib (400 mg oral BD) *vs* paclitaxel (90 mg/m^2^) + placebo (400 mg oral BD) | | PFS | | PFS in patients with/without PIK3CA/AKT1/PTEN alterations, OS ORR, CBR, DOR safety, quality of life |
| Narayan  2020 | A randomized, placebo-controlled, double-blinded, two-arm trial | | | Intervention: 451  Control:  451 | Intervention: 55 (20-82)  Control: 56 (26-86) | Patients histologically confirmed locally advanced or mTNBC, with ECOG PS 0-2, had not received prior chemotherapy for metastatic breast cancer | NR | | Nab-paclitaxel (100 mg/m^2^) + atezolizumab (80 mg) *vs* nab-paclitaxel (100 mg/m^2^) + placebo (80 mg) | | PFS, OS | | ORR, DOR, PRO and health-related quality of life |
| Lu  2021 | A randomized trial | | | Intervention: 51  Control:  51 | Intervention: 52.22±3.21  Control:  52.13±3.13 | Patients with life expectancy> 3 months, had been histologically confirmed advanced TNBC | NR | | Docetaxel (75 mg/m^2^) + oxaliplatin (135 mg/m^2^) *vs* tegafur (40-60 mg) + oxaliplatin (135 mg/m^2^) | | ORR | | CRR, DCR |
| Li  2021 | A randomized trial | | | Intervention: 57  Control:  57 | Intervention: 55.11 (50-53)  Control:  55.05 (49-52) | Patients histologically confirmed advanced TNBC | NR | | Paclitaxel (150 mg) + capecitabine (950 mg) *vs* 5-fluorouracil (500 mg, oral) + adriamycin (50 mg) + cyclophosphamide (500 mg) | | ORR | | CRR, DCR |
| Miles  2021 | A global, randomized, placebo-controlled, double-blind, phase III trial | | | Intervention: 431  Control:  220 | Intervention: 54 (22-85)  Control: 53 (25-81) | Male or female patients with metastatic or unresectable locally advanced measurable TNBC had received no prior chemotherapy or targeted therapy for TNBC and had completed any prior (neo)adjuvant chemotherapy for early breast cancer> 12 months starting the trial | Treatment continued until disease progression, unacceptable toxicity or consent withdrawal | | Paclitaxel (90 mg/m^2^) + atezolizumab (840 mg) *vs* paclitaxel (90 mg/m^2^) + placebo (840 mg) | | PFS | | OS, ORR, quality of life |
| Goldstein  2021 | A randomized, placebo controlled, double-blind phase II trial | | | Intervention: 62  Control:  61 | Intervention:  57 (29-79) Control:  57.5 (33-77) | Women ≥ 18 years old histologically confirmed measurable mTNBC, with ECOG PS ≤ 1, an adequate hematologic and organ function, were eligible to receive paclitaxel monotherapy and had received no prior systemic therapy for advanced disease | Treatment continued until disease progression | | Paclitaxel (80 mg/m^2^) + reparixin (oral 1200 mg t.i.d.) *vs* paclitaxel (80 mg/m^2^) + placebo (oral 1200 mg t.i.d.) | | PFS | | OS, ORR and safety |
| Brufsky  2021 | A randomized, multicenter, three-cohort, phase II trial | | | Intervention: 47  Control:  43 | Intervention: 55 (34-73) Control:  53 (31-80) | Patients histologically confirmed mTNBC, with ECOG PS 0/1, adequate organ and bone marrow function, and measurable disease, had a treatment-free interval from neoadjuvant/adjuvant setting≥ 6 months before start of study treatment and have prior chemotherapy, hormonal, targeted or investigational therapy | Treatment continued until disease progression or unacceptable toxicity | | Paclitaxel (80 mg/m^2^) + cobimetinib (60 mg) *vs* paclitaxel (80 mg/m^2^) + placebo | | PFS, ORR | | safety and tolerability |
| Dent  2021 | A randomized, placebo-controlled,  double-blind, phase II trial | | | Intervention: 62  Control:  62 | Intervention: 54  Control:  53 | Women≥ 18 years old with ECOG PS 0/1, had been histologically confirmed measurable locally advanced or mTNBC, and they were not amenable to curative resection if they had received no prior systemic therapy for advanced or metastatic disease, but Prior (neo) adjuvant chemotherapy and/or radiotherapy was permitted if completed≥ 6 months before the first dose | Treatment continued until disease progression or unacceptable toxicity | | Paclitaxel (80 mg/m^2^) + ipatasertib (400 mg) *vs* paclitaxel (80 mg/m^2^) + placebo (400 mg) | | OS | | Immunohistochemistry PTEN-low and PI3K/AKT pathway-activated populations |
| Annelot  2021 | A randomized, multicenter, open-label, phase 2b trial. | | | Intervention: 15  Control:  15 | Intervention:  50 (46–58.5) Control:  51 (46–60) | Patients had been histologically confirmed locally advanced TNBC or mTNBC | Treatment continued until disease progression or death | | Paclitaxel (90 mg/m^2^) + bevacizumab (10 mg/kg) *vs* paclitaxel (90 mg/m^2^) | | PFS | | OS, toxicity |
| Emens  2021a | A global, randomized, double-blind phase III trial | | | Intervention: 451  Control:  451 | Intervention: 55 (20-82)  Control:  56 (26-86) | Patients > 18 years old histologically confirmed unresectable locally advanced or mTNBC, with measurable specimen, ECOG PS 0-1, life expectancy> 3 months, qualified inspection indicators, have no contraindications for taxanes monotherapy and prior (neo) adjuvant chemotherapy was not permitted | Treatment continued until disease progression or unacceptable toxicity | | Nab-paclitaxel (100 mg/m^2^) + atezolizumab (840 mg) *vs* nab-paclitaxel (100 mg/m^2^) + placebo (840 mg) | | PFS, OS | | ORR, DOR, quality of life |
|  |  | | |  |  |  |  | |  | |  | |  |
| Emens  2021b | A randomized, double-blind phase III trial | | | Intervention: 451  Control:  451 | NR | Patients ≥ 18 years old histologically confirmed unresectable locally advanced measurable or mTNBC, with ECOG PS 0-1, had received no prior chemotherapy or targeted therapy for TNBC and had completed any prior (neo) adjuvant chemotherapy for early breast cancer> 12 months starting the trial | Treatment continued until disease progression or toxicity | | Nab-paclitaxel (100 mg/m^2^) + atezolizumab (840 mg) *vs* nab-paclitaxel (100 mg/m^2^) + placebo (840 mg) | | PFS, OS | | NR |
| Duan  2021 | A randomized trial | | | Intervention: 56  Control:  56 | Intervention: 50.53±5.85  Control: 50.47±6.22 | Patients had been histologically confirmed advanced TNBC | NR | | Abraxane (260 mg/m^2^) + oxaliplatin (100 mg/m^2^) *vs* abraxane (260 mg/m^2^) | | ORR | | CRR, DCR, quality of life |

**Abbreviation:** mTNBC, metastatic triple negative breast cancer; ECOG PS, Eastern Cooperative Oncology Group performance status; NR, none report; PFS, progression-free survival; OS, overall survival; CRR, complete response rate; ORR, objective response rate; DCR, disease control rate; CBR, Clinical Benefit Response; DOR, duration of response;
