# Supplementary figures and images for: Efficacy and safety of taxanes combined with chemotherapy drugs in advanced triple negative breast cancer: A meta-analysis of 26 randomized controlled trials
Source: Front Oncol. 2022 Aug 31;12:972767. doi: 10.3389/fonc.2022.972767 (PMC9471016; doi:10.3389/fonc.2022.972767)

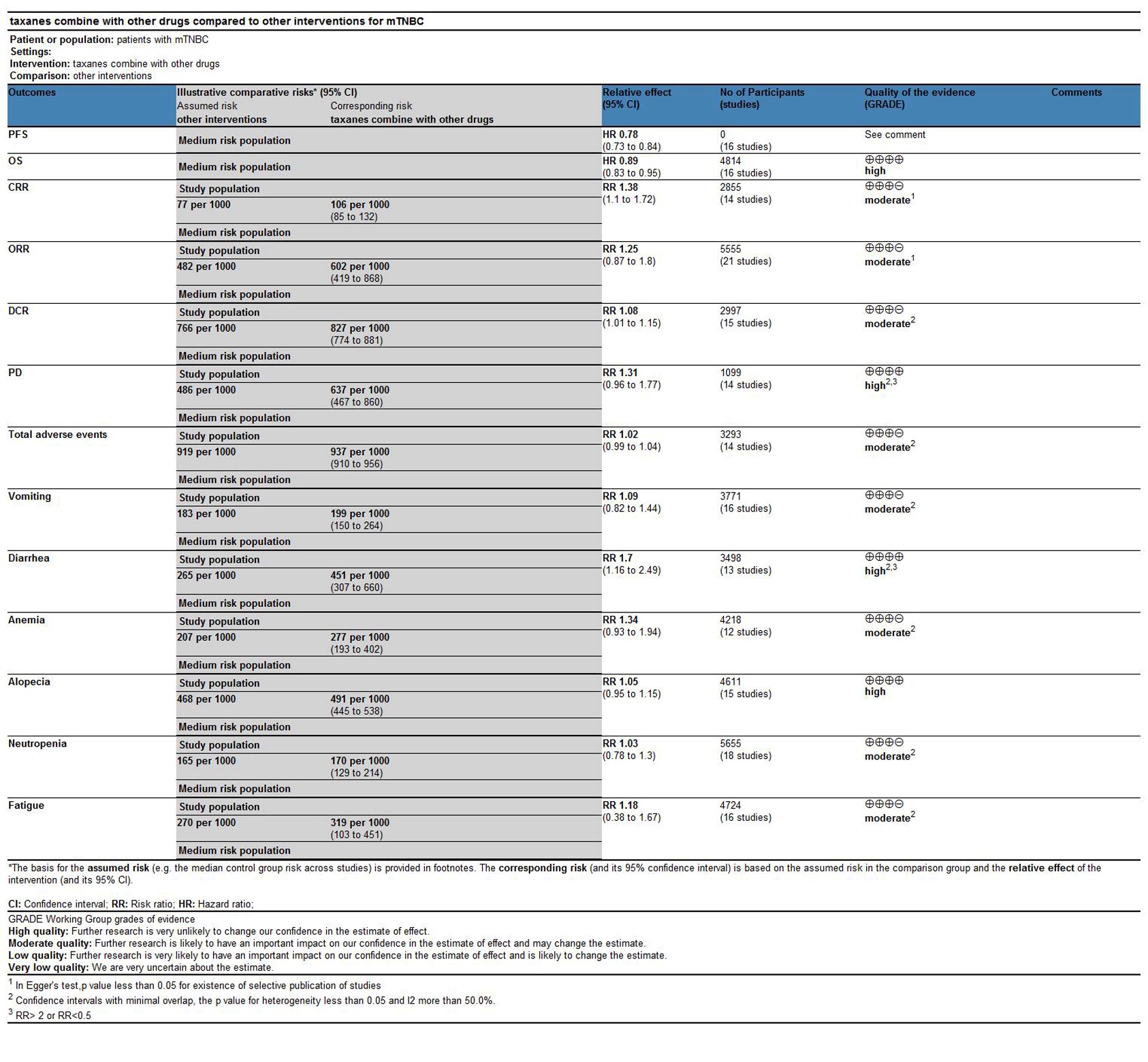

Supplement: Supplementary file 3 [file Table_3.docx]
